# Pterodontic Acid Isolated from *Laggera pterodonta* Inhibits Viral Replication and Inflammation Induced by Influenza A Virus

**DOI:** 10.3390/molecules22101738

**Published:** 2017-10-16

**Authors:** Wenda Guan, Jing Li, Qiaolian Chen, Zhihong Jiang, Rongping Zhang, Xinhua Wang, Zifeng Yang, Xiping Pan

**Affiliations:** 1 Tropical Medicine Institute, Guangzhou University of Chinese Medicine, Guangzhou 510405, China; guanwenda2004@163.com; 2 State Key Laboratory of Respiratory Disease, National Clinical Research Center for Respiratory Disease, First Affiliated Hospital of Guangzhou Medical University, (Guangzhou Medical University), Guangzhou 510120, China; lijinghenan@163.com (J.L.); qiangwei86@163.com (Q.C.); 3 State Key Laboratory of Quality Research in Chinese Medicine, Macau University of Science and Technology, Macau (SAR) 519020, China; zhihongjiang@gmail.com; 4 School of Pharmaceutical Science & Biomedical Engineering Research Center, Yunnan Key Laboratory of Pharmacology for Natural Products, Kunming Medical University, Kunming 650500, China; zhrpkm@163.com; 5 Institute of Chinese Integrative Medicine, Guangzhou Medical University, Guangzhou 511436, China

**Keywords:** *Laggera pterodonta*, pterodontic acid, influenza A virus, antiviral activity, anti-inflammation

## Abstract

*Laggera pterodonta* (DC.) Benth. is a traditional Chinese medicine. The previous study revealed that the crude extracts of this herb could inhibit influenza virus infection, but its anti-influenza components and underlying mechanism of action remain unknown. Column chromatography was performed to isolate components from the plant. Activity against influenza virus of the compound was determined by CPE inhibition assay. Neuraminidase (NA) inhibition was measured by chemiluminescence assay. The anti-virus and anti-inflammation effects were determined using dual-luciferase reporter assay, immunofluorescence, quantitative real-time PCR and luminex assay. Pterodontic acid was isolated from *L. pterodonta*, which showed selective anti-viral activities to H1 subtype of human influenza A virus. Meanwhile, the NA activity was not obviously inhibited by the compound. Further experiments exhibited that the compound can suppress the activation of NF-κB signal pathway and export of viral RNP complexes from the nucleus. In addition, it can significantly attenuate expression of the pro-inflammatory molecules IL-6, MIP-1β, MCP-1, and IP-10 induced by human influenza A virus (H1N1) and similarly downregulate expression of cytokines and chemokines induced by avian influenza A virus (H9N2). This study showed that in vitro antiviral activity of pterodontic acid is most probably associated with inhibiting the replication of influenza A virus by blocking nuclear export of viral RNP complexes, and attenuating the inflammatory response by inhibiting activation of the NF-κB pathway. Pterodontic acid might be a potential antiviral agent against influenza A virus.

## 1. Introduction

*Laggera pterodonta* (DC.) Benth. (Compositae) is a perennial herb widely distributed in southwest China, especially in Yunnan province. Its aerial part has been used as a folk medicine for treating respiratory virus infection, sore throat, bronchitis, and fever [1,2]. So far, dozens of compounds have been isolated from this plant. Among them sesquiterpenoid and flavonoid are the main types [3]. Most sesquiterpenoids of *L. pterodonta* have an eudesmane skeleton and possess wide range of pharmacological activities. For instance, pterodontriol A, pterodontic acid, and ilicic acid have inhibitory effect on tumor cells [4]. Pterodontic acid has an anti-inflammatory effect as observed by decreasing xylene induced ear edema in mice [5].

Epidemics and pandemics caused by influenza viruses have resulted in mass deaths worldwide. The 1918 flu influenza pandemic resulted in death of 50–100 million (three to five percent of the world’s population) [6]. Furthermore, with the emergence of the H5N1, H1N1pdm09, H7N9, H5N6 highly pathogenic avian influenza viruses (HPAIV), as well as the rapid evolution of the virus via mutation to evade the current control measures of vaccination and antiviral drugs (M2 ion channel blockers, neuraminidase inhibitors), influenza remains one of the major health threats to public health [7,8]. Therefore, it is necessary to develop new antiviral agents that are less susceptible to virus mutation and resistance.

One of the new strategies in the field of antiviral drug discovery is to exploit host innate antiviral factors and mechanisms to counter viral infections. The influenza virus infection induced-innate immunity leads to the activation of nuclear factor kappa B (NF-κB) induced the production of pro-inflammatory cytokines and chemokines (for example, interleukin (IL-6, TNF-α, MCP-1, MIP-1α/β, and CCL-5)). High morbidity and mortality from influenza virus infection is correlated with overproduction of pro-inflammatory cytokines (cytokine storm) [9,10]. It is shown that inhibiting the host’s immune response against influenza virus using an immunomodulatory drug provides significant protection from mortality. Besides the drugs are less susceptible to virus resistance because they inhibit inflammation, but not influenza virus protein (M2, NA) [11]. As a result, dampening host innate immune-mediated pulmonary injury has been a rational treatment strategy to influenza virus infection.

Many herbal extracts and natural products of traditional Chinese medicine (TCM) have extensive antiviral activities, including suppression of various influenza virus subtypes and other respiratory viruses. *L. pterodonta* is one of commonly used antiviral TCM materials. Its crude extract has been developed into several drug formulations for antiviral use in China. It has been reported that the extracts and flavonols from *L. pterodonta* exhibited antiviral activities against respiratory syncytial virus (RSV), herpes simplex type I (HSV-I), herpes simplex type II (HSV-II), enterovirus 71 (EV71), respectively [12,13]. Our previous biological evalution also suggested that the sesquiterpene fraction of *L. pterodonta* had an anti-influenza virus effect [14]. Pterodontic acid is one of the main sesquiterpenoids from *L. pterodonta*, its effect on influenza virus has not been reported before. In this study, the anti-influenza activity of pterodontic acid is evaluated using cytopathic effect (CPE) reduction assay. Its action mechanism on influenza virus and host immune response is explored further.

## 2. Results

### 2.1. Identification of Pterodontic Acid by NMR

**Figure 1:**
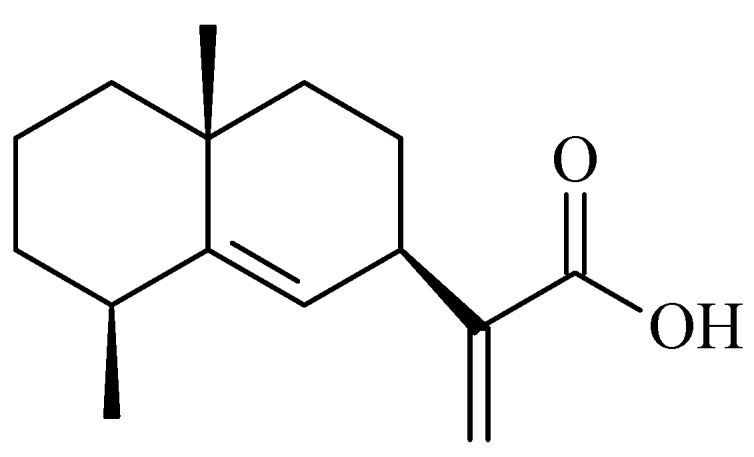
Chemical structure of pterodontic acid.

White powder, ^1^H-NMR (400 MHz, CDC1_3_) ppm: 6.31 (1H, br s, H-13c), 5.68 (1H, br s, H-13t), 5.20 (1H, br s, H-6), 1.16 (3H, s, H-14), 1.18 (3H, d, *J* = 8.0 Hz, H-15), 3.32 (1H, m, H-7), 2.47 (1H, m, H-4), 2.0~1.4 (10H, m, H-1,2,3,8,9); ^13^C-NMR (100 MHz, CDCl_3_) ppm: 42.87 (C-1), 17.52 (C-2), 33.20 (C-3), 38.14 (C-4), 144.90 (C-5), 122.80 (C-6), 38.20 (C-7), 26.60 (C-8), 41.51 (C-9), 34.41 (C-10), 149.14 (C-11), 171.93 (C-12), 125.76 (C-13), 27.22 (C-14), 23.19 (C-15). The data was accordance with document of pterodontic acid (Figure 1) [15]. 

### 2.2. Cytotoxicity In Vitro

**Figure 2:**
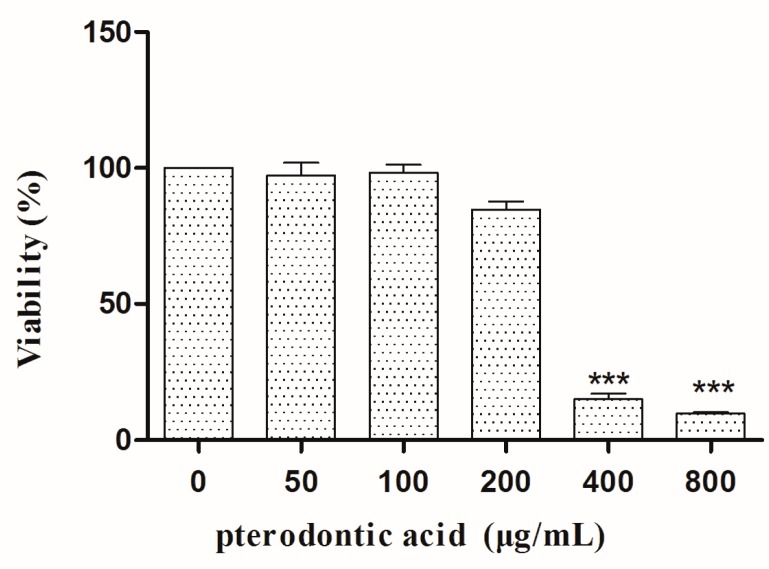
Cytotoxic effect of pterodontic acid on MDCK cells. For pterodontic acid cytotoxicity assays, MDCK cells were incubated with different concentrations of the compound. After 48 h, cell viability was measured by MTT assay. Values represent the mean (%) ± SD from three independent experiments. An ANOVA with Tamhane’s post-hoc analysis was applied, *** *p* < 0.001, relative to the values of untreated cells.

After 48 h incubation, the MTT assay showed that the concentration required for 50% cytotoxicity (TC_50_) of pterodontic acid was 278.9 μg/mL (Figure 2).

### 2.3. Anti-Viral Activity In Vitro

**Figure 3:**
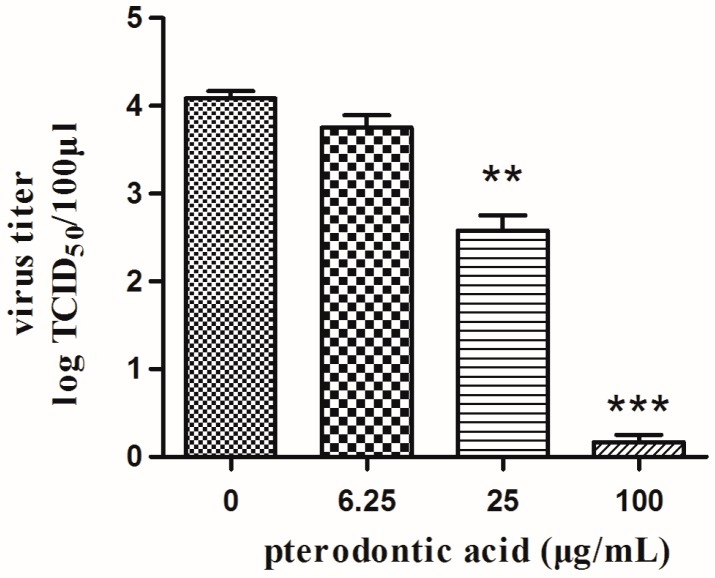
Assay of antiviral activity of pterodontic acid by progeny virus reduction assay. MDCK cells infected with 100 TCID_50_A/PR/8/34 (H1N1) in the absence or presence of pterodontic acid and supernatants was collected at 24 h post-infection. The progeny viruses from MDCK cells supernatants were determined by CPE assay in MDCK cells. Data were expressed as mean ± SD from three separate experiments. Data were obtained from three separate experiments. ** *p* < 0.01; *** *p* < 0.001; compared with influenza virus-infected alone.

Pterodontic acid showed different magnitudes against a serial of influenza viruses subtypes with IC_50_ values of 9.47–37.14 μg/mL and SI values of 7.51–29.45 (Table 1). In addition, the progeny virus titers were obviously decreased in a dose-dependent manner by the compound (Figure 3).

### 2.4. Activities on Neuraminidase 

Pterodontic acid was investigated for inhibitory effect on NAs, which were from A/PR/8/34 (H1N1), A/California/04/2009 (H1N1) and A/GZ/GIRD02/09 (H1N1). The compound showed mild inhibitory activity only for A/California/04/2009 (H1N1), and the IC_50_ value was much lower than oseltamivir (Table 2). 

### 2.5. Suppression of NF-κB Signaling Pathway Activation

**Figure 4:**
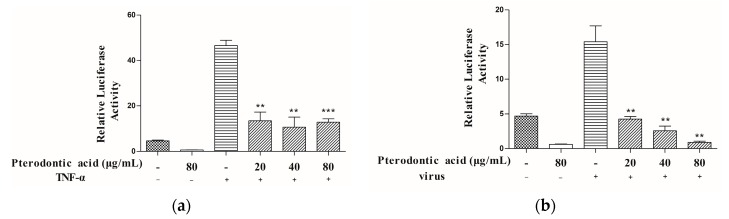
Effects of pterodontic acid on TNF-α and influenza virus-induced activation of NF-κB pathway. HEK293 cells were stably transfected with NF-κB luciferase reporter plasmid. (**a**) The cells were stimulated with 20 ng/mL TNF-α in the presence or absence of different concentration of pterodontic acid for 24 h; (**b**) The cells were stimulated with influenza A virus (MOI = 0.1) in the presence or absence of different concentration of pterodontic acid for 24 h. After extraction of protein from HEK293 cells, the expression of NF-κB was assayed through luciferase activity. ** *p* < 0.01; *** *p* < 0.001, relative to TNF-α or virus control.

NF-κB is a key pathway of the innate immune response to viral infections and is regarded as a suitable cellular target. Inhibition NF-κB activity is virtually resistant to infection with influenza virus [16]. The pathway may affect the virus replication cycle and regulate excessive inflammation due to accumulation of influenza viral RNA species and over-expression of viral hemagglutinin, nucleoprotein or matrix proteins [17]. Thus, we used a NF-κB-driven luciferase reporter gene to evaluate pterodontic acid’s effect on activation of NF-κB. The results of this assay showed pterodontic acid inhibited NF-κB activation stimulated by influenza virus in a dose-dependent manner with a range from 20 μg/mL to 80 μg/mL, and NF-κB activation was stimulated by TNF-α (Figure 4).

### 2.6. Inhibition of Viral RNP Export

**Figure 5:**
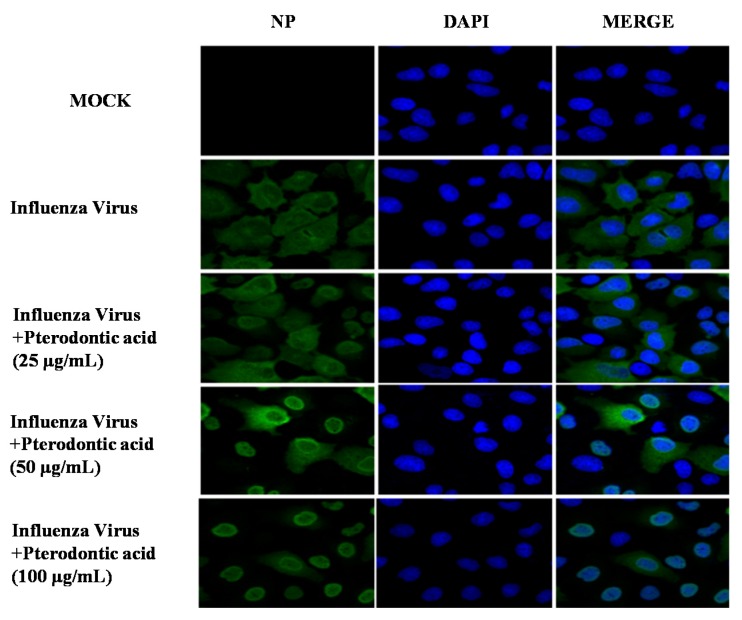
Pterodontic acid inhibited nuclear export of viral RNP. A549 cells infected with A/PR/8/34 (H1N1) virus (MOI = 0.1) in the absence or presence of different concentration of pterodontic acid for 8 h. The cells were stained with anti-viral nuclear protein antibody labeled with fluorescent monomer (green).The nucleus was stained with DAPI (blue).

Influenza virus infection induces NF-κB transcription factor resulting in activation of caspases through expression of proapoptotic factor, FasL, and TRAIL [18,19]. It shows that inhibitors or siRNA of caspase impaired influenza virus propagation due to retention viral RNP complexes in the nucleus [20]. To determine whether the inhibition of NF-κB signal pathway influences the nuclear export of viral RNP, an anti-viral nuclear protein antibody and DAPI were used to stain viral nucleoprotein (NP) and nucleus of cell, respectively. Immunofluorescence showed pterodontic acid obviously inhibited the nuclear export of viral RNP in a significant dose–response manner (Figure 5). The result implied that pterodontic acid reduced virus propagation due to blocking the influenza viral RNP export induced by downregulating expression level of the NF-κB pathway.

### 2.7. Impact on Influenza Associated Cytokines Expression in Infected A549 Cells

**Figure 6:**
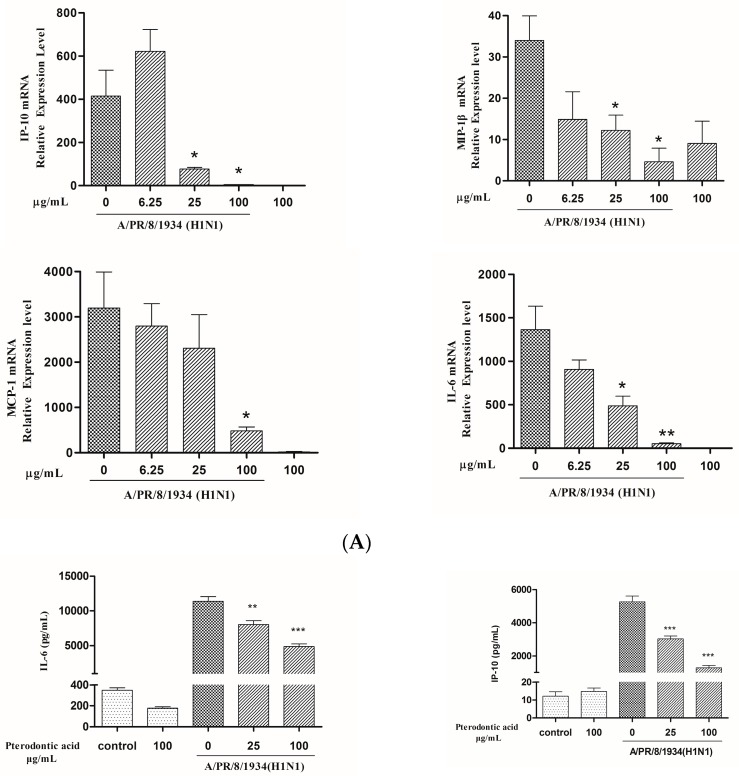

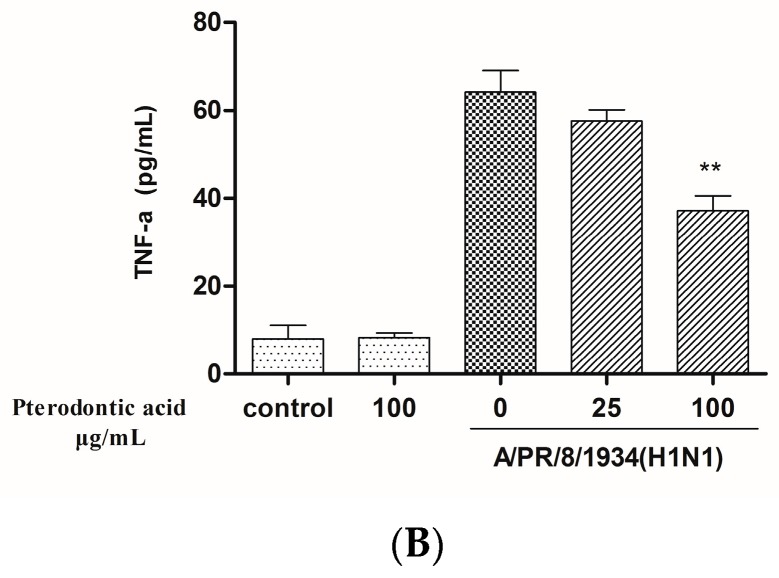
Pterodontic acid inhibited pro-inflammatory expression in influenza virus A/PR/8/34 (H1N1)-infected A549 cells. (**A**) A549 cells were infected with influenza virus (MOI = 0.2) in the presence or absence of different concentration of pterodontic acid for 24 h. Then, Total RNA was extracted and measured by qRT-PCR for the expression levels of IL-6, IP-10, MIP-1β, and MCP-1. The expression of each target gene was normalized to GAPDH; (**B**) The protein levels of cytokines and chemokines in the culture supernatant were assayed by luminex 24 h post A/PR/8/34 infection alone or in combination with pterodontic acid at 25 and 100 µg/mL. Each experiment was repeated in triplicate. * *p* < 0.05, ** *p* < 0.01, *** *p* < 0.001, relative to virus control.

**Figure 7:**
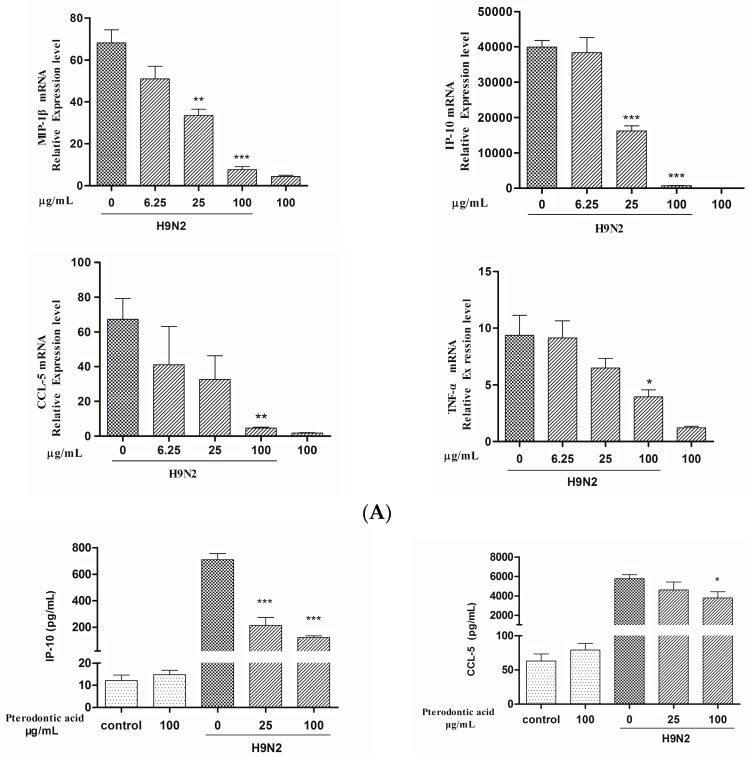

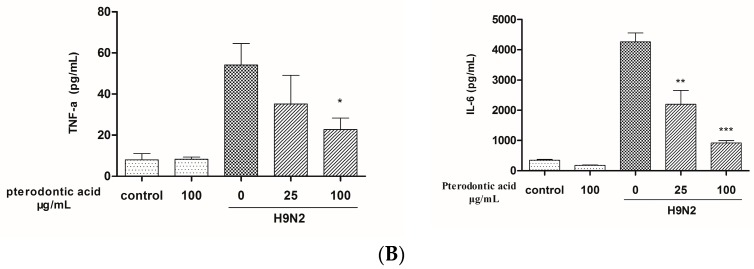
Pterodontic acid inhibited pro-inflammatory expression in influenza virus A/Chicken/Guangdong/1996/ (H9N2)—infected A549 cells. (**A**) A549 cells were infected with influenza virus (MOI = 0.2) in the presence or absence of different concentration of pterodontic acid for 24 h. Then, Total RNA was extracted and measured by qRT-PCR for the expression levels of CCL-5, IP-10, TNF-α, and MIP-1β. The expression of each target gene was normalized to GAPDH; (**B**) The protein levels of cytokines and chemokines in the culture supernatant were assayed by luminex 24 h post A/Chicken/Guangdong/1996/ (H9N2) infection alone or in combination with pterodontic acid at 25 100 µg/mL. Each experiment was repeated in triplicate. * *p* < 0.05, ** *p* < 0.01, *** *p* < 0.001, relative to virus control.

Excessive inflammation due to overabundant production of pro-inflammatory cytokines and chemokines by airway epithelial cells (cytokines storm) is considered as an important factor of deaths caused by influenza virus. The NF-κB pathway plays a major role in regulating expression of pro-inflammatory genes in inflammation caused by various factors, such as cancer, bacteria, virus, etc. [21]. Pterodontic acid has shown significantly inhibited influenza virus-induced activation of NF-κB. To identify effects on pro-inflammatory genes, the levels of mRNA and protein of cytokines and chemokines associated with influenza A virus H1N1 infection or treated with pterodontic acid in A549 cells were detected by quantitative real-time PCR and Luminex. The result showed that pterodontic acid significantly decreased the expression of IP-10, MIP-1β, MCP-1, and IL-6 in A549 cells infected by human influenza A virus (H1N1) (PR8 strain)(Figure 6), as well as the expression of CCL-5, IP-10, MIP-1β, and TNF-α in A549 cells infected by avian influenza A virus (H9N2) (Figure 7). The concentration of pterodontic acid was in a dose-dependent manner with above cytokines and chemokines. These data indicate that pterodontic acid inhibited NF-κB activation-inducing proinflammatory responses associated with human or avian influenza A virus infection.

## 3. Discussion

Although several drug formulations containing crude extracts of *L. pterodonta* have been used to treat respiratory infection in China for many years, the antiviral components of *L. pterodonta* remain unknown. The quality standards of these drugs lack reliable chemical marker. In this study, a major eudesmane sesquiterpenoid was isolated from *L. pterodonta* and identified as pterodontic acid, which was unambiguously proved to be an effective anti-influenza virus component by CPE inhibition, pro-inflammatory cytokine, and chemokine assays for the first time. Therefore, pterodontic acid may be used as a chemical marker for quality control of *L. pterodonta*-driven drugs.

In order to explore the anti-influenza viral and anti-inflammatory mechanism of pterodontic acid, its inhibitory effects on influenza virus proteins (NA, vRNP) and activation of the pathway inducing pro-inflammatory cytokine expression were tested. The results showed that pterodontic acid could markedly suppress export of viral RNP complexes from the nucleus, while exhibiting only a weak inhibitory effect on NA activity. It can be concluded that pterodontic acid reduces influenza viral titer by affecting vRNP export, rather than by affecting NA activity. The results also showed that pterodontic acid could suppress activation of NF-κB in H1N1 virus or TNF-α-stimulated HEK-293T cells. NF-κB is known to be a main pro-inflammatory pathway which can be activated by virus infection [22]. Since virus-induced NF-κB activation can enhance influenza virus RNP export through promoting expression of caspase [19,20], NF-κB may be a key factor for pterodontic acid to inhibit vRNP export and expression of pro-inflammatory cytokine.

As a potential therapeutic target in influenza virus infection, NF-κB not only influences pathogenesis of influenza virus by direct effects on the virus life cycle, but also induces production of pro-inflammatory cytokines (IL-1, IL-6, IL-18, and TNF-α) and chemokines (RANTES, MIP-1α/β, MCP-1, MCP-3, and IP-10) [17,23]. There is evidence that an imbalanced overproduction of cytokines and chemokines (cytokine storm) depending on NF-κB signaling function can cause the severe lung damage of HPAIV [24,25]. A previous study confirmed that inhibition of NF-κB greatly reduced viral titers and expression of cytokines and chemokines in vitro and in vivo [26]. Similarly, our results showed that pterodontic acid reduced expression level of IL-6, IP-10, MIP-1β, and MCP-1 through inhibiting activation of NF-κB induced by H1N1 influenza A virus, and downregulated production of CCL-5, IP-10, TNF-α, and MIP-1β in H9N2 influenza A virus infection. This implied that anti-viral activity of pterodontic acid was associated with reducing the release of both pro-inflammatory cytokines and chemokines. Therefore, pterodontic acid might be a potential lead compound for new anti-flu drug development.

## 4. Materials and Methods

### 4.1. Reagents and Materials

Column chromatography (CC) was performed using silica gel (200–300 mesh, Qingdao Haiyang, Qingdao, China) and TLC was performed on pre-coated silica gel GF254 plates (Qingdao Haiyang). Spots were visualized under UV light (254 or 365 nm) or using iodine fuming. All solvents used were of analytical grade (Guangzhou Chemical Reagents Company, Ltd., Guangzhou, China). MTT and DMSO were purchased from the Sigma-Aldrich Chemicals Co. (St. Louis, MO, USA). A monoclonal antibody against nucleoprotein (NP) of influenza A virus was brought from Abcam (Cambridge, MA, USA). Recombinant human cytokine tumor necrosis factor alpha (TNF-α) was purchased from R&D system (Minneapolis, MN, USA). 4′,6-diamidino-2-phenylindole (DAPI) was purchased from Roche Applied Science (Basel, Switzerland). The bright-Glo luciferase assay detection kit was purchased from Promega (Madison, WI, USA).

Human alveolar epithelial cell line (A549), Madin–Darby Canine Kidney (MDCK) cells were from the American Type Culture Collection. HEK293 cells, stably co-transfected with pNF-κB-TATA-F-LUCI and pQCXIP-eGFP plasmids were provided by Dr. Li Chufang from the State Key Laboratory of Respiratory Disease. Influenza virus A/PR/8/1934 (H1N1) (PR8 strain) and influenza virus A/Aichi/2/1968 (H3N2) (Aichi strain) were kindly provided by Dr. Zhang Fengxue from Guangzhou University of Chinese Medicine. Avian influenza A viruses H6N2/H7N3/H9N2 isolated from chicken were supplied by Dr. Chen Jianxin from South China Agriculture University. Influenza virus A/California/04/2009 (H1N1) (pdm09 H1N1 strain) was a gift from Dr. Chris Mok in Hong Kong University and propagated in embryonated chicken eggs. A/GZ/GIRD02/09 (H1N1) was isolated from human in our laboratory. Viral titers were measured by a 50% tissue culture infectious dose (TCID_50_) assay in relevant cells.

### 4.2. Plant Material and Preparation of Pterodontic Acid

*L. pterodonta* was collected from Yunnan province. The herbarium specimen was authenticated by Professor Rongping Zhang and deposited in the College of Pharmaceutical Sciences, Kunming Medical University.

The powdered plant material (1 kg) was extracted with methanol using a percolation process, followed by collecting a 40 L elution and vacuum-concentrating to yield a 135 g methanol extract. The extract was suspended in H_2_O (800 mL) and subjected to liquid–liquid partition by adding petroleum ether. The residue (48 g) of the petroleum ether layer was subjected to silica gel CC (petroleum ether–EtOAc, 10:1) to obtain fraction A (38 g). Fraction A (8 g) was subjected to silica gel CC with elution by a gradient of petroleum ether–EtOAc (1:0; 20:1; 10:1; 5:1; 2:1) to yield fractions 1–12 based on TLC analysis. Fr.1 (3.8 g) was the petroleum ether elution and main elution of Fraction A. Fr.1 was further subjected to silica gel CC (petroleum ether–CHCl_3_, from 100:0 to 95:5) to obtain five fractions (I–V) for TLC analysis, Fr.III was further purified by gel CC to afford a compound (34.7 mg) with purity higher than 95%, which was identified as pterodontic acid (Figure 1).

### 4.3. In Vitro Cytotoxicity Assay

The cytotoxicity of pterodontic acid in MDCK cells was tested by the MTT assay as follows: MDCK cells were dispensed into 96-well plates (1 × 10^5^ cells/well) for overnight culture. Varying concentrations of pterodontic acid were added to appropriate wells for 48 h of incubation. Each concentration of pterodontic acid was assessed in triplicate wells. After incubation, the culture medium was replaced by filtered MTT solution (0.5 mg/mL). The plates were incubated for 4 h at 37 °C. The formazan crystals were dissolved in 200 μL of DMSO after supernatant removal. The absorbance was measured in a spectrophotometer (Molecular Devices, San Francisco, CA, USA) at a wavelength of 570 nm for TC_50_ calculation.

### 4.4. In Vitro Antiviral Assayand Progeny Virus Reduction Assay

A cytopathic effect (CPE) inhibition assay was conducted using MDCK cells infected with serial influenza virus strains. Cells (2.5 × 10^4^ cells/well) were seeded in 96-well plates and incubated overnight at 37 °C under 5% CO_2_. Then 100 TCID_50_ of virus was added and incubated at 34 °C for 2 h after culture medium removal. Infected cells were washed and cultured in the presence of varying concentration of pterodontic acid or ribavirin (5–400 μM) in MEM supplemented with 2 μg/mL TPCK-trypsin (Sigma, St. Louis, MO, USA). Each concentration of drugs was tested in triplicate wells. After 72 h incubation, the CPE were observed by microscopy and the IC_50_ of drugs was calculated using the Reed–Muench method as in a previous study [27]. The selectivity index (SI) was equal to the ratio of TC_50_/IC_50_.

Progeny virus reduction assay: A confluent monolayer of MDCK cells grown in a 96-well plate was washed twice with phosphate buffered saline (PBS) and then inoculated with 10-fold serial dilutions of the virus-containing supernatants in DMEM for 2 h at 37 °C. The inoculum was removed and cells were incubated with 200 μL DMEM containing 1.5 μg/mL TPCK-trypsin. After 48 h of incubation, the cytopathic effect (CPE) in virally infected cells was observed microscopically, and the TCID_50_ was determined using methods described by Reed and Muench [28].

### 4.5. NA Activity Assay

A chemiluminescent assay was used to measure the resistance level of influenza virus isolates to pterodontic acid activity. The chemiluminescent neuraminidase substrate was cleaved by NA to yield a chemiluminescent product that could be quantified. A NA-Star Influenza Neuraminidase Inhibitor Resistance Detection Kit (Applied Biosystems, Foster City, CA, USA) was appled in the test. The different virus strains were 1:5 diluted using NA-Star assay buffer and injected with 25 µL dilution virus (or not) per well. Serial dilutions (10 half-log dilutions) of the compound in the NA-Star assay buffer was added at 25 µL (or not) per well. The mixture was incubated at 37 °C for 10–20 min. After incubation, 10 µL of NA-Star substrate was injected into each well and incubated at 37 °C for 10–30 min. Then, 60 µL of NA-Star accelerator was injected into each well and measured with a luminometer (Thermo Scientific Varioskan Flash, Vantaa, Finland). The IC_50_ of the compound was calculated using GraphPad Prism.

### 4.6. NF-κB Reporter Assay 

The impact of pterodontic acid on NF-κB transcriptional activity was detected as follows: HEK293 cells, which were stably co-transfected with pNF-κB-TATA-F-LUCI and pQCXIP-eGFP plasmid, were incubated in a 96-well plate (5 × 10^4^ cells/well). Cell medium was replaced by serum-free DMEM including TNF-α (R&D systems) or 0.1 MOI of influenza virus (A/PR/8/34, H1N1), with or without pterodontic acid incubation for 24 h, respectively. A luciferase assay system (Promega) was employed to measure luciferase activities normalized by the level of GFP expression.

### 4.7. Immunofluorescence Staining

The location of the nucleoprotein (NP) of influenza A virus was detected by immunofluorescence in A549 cells as follows: the influenza virus infected cells, treated or not treated with pterodontic acid, were fixed in 4% paraformaldehyde at room temperature for 30 min. The cells were incubated with blocking solution (5% FBS) for 30 min after treatment with 1%Triton X-100 for 15 min. Mouse anti-NP antibody (1:1000) (Abcam) (primary antibody) and a FITC-labelled goat anti-mouse IgG (1:150) (secondary antibody) were used to detect nucleoprotein. Observation was conducted with a fluorescence microscope (Zeiss Axiovert 135, Zeiss, Oberkochen, Germany).

### 4.8. mRNA Expression of Cytokines Assay by qRT-PCR

A549 cells were seeded into 24-wells plates (1 × 10^5^ cells/well) and incubated overnight at 37 °C under 5% CO_2_. Cells were infected with 100 TCID_50_ of influenza virus (PR8 and H9N2 respectively) at 34 °C for 2 h. Infected cells were washed and cultured with a two-fold serial dilution of pterodontic acid (25, 100 μg/mL). After 24 h incubation, nucleic acid was extracted from cells using an RNeasy Mini Kit (Qiagen, Austin, TX, USA) and the mRNA expression of influenza associated cytokines (IL-6, MCP-1, IP-10, TNF-α, CCL-5, MIP-1α) (Table 3) were detected by a one-step real-time PCR using a one-step primescript RT-PCR kit (Perfect real time) (TaKaRa, Mountain View, CA, USA). The reaction conditions were as follows: 95 °C 15 s, 55 °C 35 s for 40 cycles using 7500 Real time PCR (ABI, Scottsdale, AZ, USA).The relative quantification of PCR products was calculated according to a previous study [27].

### 4.9. Measurement of Protein Level of Cytokine

Cell-culture supernatants were collected and cytokine concentrations in the supernatants were measured with luminex using a procartaplex multiplex immunoassay kit (eBioscience, ThermoFisher, Waltham, MA, USA) according to the manufacturer protocol.

### 4.10. Statistical Analysis 

The results were expressed as the mean ± SD for three independent experiments. Statistical analysis of data was performed using one-way analysis of variance (ANOVA) followed by student Newman–Keuls test to evaluate difference among multiple groups. Differences were considered significant at *p* values less than 0.05.

## 5. Conclusions

This study showed that pterodontic acid, one of the major sesquiterpenoids of *L. pterodonta*, had powerful in vitro activities of anti-influenza A virus. Furthermore, the compound exhibited inhibition of nuclear export of viral RNP complexes and suppression of pro-inflammatory response caused by the activation of NF-κB. It appeared to have a dual anti-virus and anti-inflammatory function. The result may help us to understand the mechanism of the anti-influenza virus of *L. pterodonta* and provide clues for developing a novel antiviral drug.

## Figures and Tables

**Table 1:** Antiviral activity of pterodontic acid against influenza viruses

	Influenza Virus Type and Strain							
	A/PR/8/34 (H1N1)	A/California/04/2009 (H1N1)	A/GZ/GIRD07/09 (H1N1)	A/Aichi/2/68 (H3N2)	A/Duck/Guangdong/1994 (H7N3)	A/Duck/Guangdong/2009 (H6N2)	A/HK/Y280/97 (H9N2)	B/Lee/1940 (FluB)
Pterodontic acid IC_50_ (μg/mL)	37.14	21.75	9.47	>278.9	>278.9	>278.9	>278.9	>278.9
SI	7.51	12.82	29.45	<1	<1	<1	<1	<1
Ribavirin IC_50_ (μg/mL)	8.84	4.42	25	39.68	19.84	50	35.36	12.5

**Table 2:** Inhibition of pterodontic acid on NAs from Influenza A viruses

	NA Inhibitory Effect (IC_50_ (μg/mL))		
	A/PR/8/34 (H1N1)	A/California/04/2009 (H1N1)	A/GZ/GIRD02/09 (H1N1)
pterodontic acid	-	668.1	-
Oseltamivir	3.12 × 10^−4^	2.54 × 10^−4^	3.41 × 10^−4^

-: No suppression.

**Table 3:** Primers and probe sequences for qRT-PCR.

Gene	Primes and Probe	Sequence (5′→3′)
*IL-6*	Forward	CGGGAACGAAAGAGAAGCTCTA
	Reverse	CGCTTGTGGAGAAGGAGTTCA
	Probe	TCCCCTCCAGGAGCCCAGCT
*IP-10*	Forward	GAAATTATTCCTGCAAGCCAATTT
	Reverse	TCACCCTTCTTTTTCAT-TGTAGCA
	Probe	TCCACGTGTTGAGATCA
*MIP-1β*	Forward	AAAACCTCTTTGCCACCAATACC
	Reverse	GAGAGCAGAAGGCAGCTACTAG
	Probe	TGAAGCTCTGCGTGACTGTCCTGTCT
*MCP-1*	Forward	CAAGCAGAAGTGGGTTCAGGAT
	Reverse	AGTGAGTGTTCAAGTCTTCGGAGTT
	Probe	CATGGACCACCTGGACAAGCAAACC
*CCL-5*	Forward	CAGCAGTCGTCTTTGTCACC
	Reverse	GTTGATGTACTCCCGAACCC
	Probe	CGCCAAGTGTGTGCCAACCC
*TNF-α*	Forward	AACATCCAACCTTCCCAAACG
	Reverse	GACCCTAAGCCCCCAATTCTC
	Probe	CCCCCTCCTTCAGACACCCTCAACC
*GAPDH*	Forward	GAAGGTGAAGGTCGGAGTC
	Reverse	GAAGATGGTGATGGGATTTC
	Probe	CAAGCTTCCCGTTCTCAGCC

## Abbreviations

IL-6: interleukin-6
MCP-1: monocytes chemotactic factor
IP-10: interferon gamma-induced protein 10
TNF-α: tumor necrosis factor alpha
NF-κB: Nuclear factor-κB
CCL-5: Chemokine (C-C motif) ligand 5
MIP-1β: Macrophage Inflammatory Proteins-1Beta
RNP: ribonucleoprotein
MTT: methylthiazolyltetrazolium
HEK-293: Human embryonic kidney 293 cells
GFP: green fluorescent protein
HPAIV: highly pathogenic avian influenza virus
HSV-I: herpes simplex type I
HSV-II: herpes simplex type II
RSV: respiratory syncytial virus
NP: nucleoprotein
